# Interdisciplinary challenges and aims of flap or graft reconstruction surgery of sinonasal cancers: What radiologists and radiation oncologists need to know

**DOI:** 10.3389/fonc.2022.1013801

**Published:** 2022-09-20

**Authors:** Florent Carsuzaa, Benjamin Verillaud, Pierre-Yves Marcy, Philippe Herman, Xavier Dufour, Valentin Favier, Juliette Thariat

**Affiliations:** ^1^Department of Oto-Rhino-Laryngology-Head and Neck Surgery, University Hospital of Poitiers, Poitiers, France; ^2^Laboratoire Inflammation, Tissus Epithéliaux et Cytokines (LITEC), University of Poitiers, Poitiers, France; ^3^Department of Oto-Rhino-Laryngology-Head and Neck Surgery, Hôpital Lariboisière, Paris, France; ^4^Department of Radiology, Clinique du Cap d’Or, La Seyne-sur-mer, France; ^5^Department of Oto-Rhino-Laryngology-Head and Neck Surgery, Hôpital Gui de Chauliac, University Hospital of Montpellier, Montpellier, France; ^6^Department of Radiation Oncology, Centre François Baclesse, Caen, France; ^7^Laboratoire de Physique Corpusculaire UMR6534 IN2P3 ENSICAEN CNRS, Normandy University, Caen, France

**Keywords:** sinonasal tumors, flap, reconstructive surgery, radiotherapy, imaging

## Abstract

In sinonasal cancer surgery, a fundamental challenge is to understand the postoperative imaging changes after reconstruction. Misinterpretation of post-operative imaging may lead to a misdiagnosis of tumor recurrence. Because radiotherapy planning is based on imaging, there are many gaps in knowledge to be filled in the interpretation of postoperative imaging to properly define radiotherapy tumor volumes in the presence of flaps. On the other hand, radiotherapy may be responsible for tissue fibrosis or atrophy, the anatomy of the reconstructed region and the functional outcomes may change after radiotherapy compared to surgery alone. This narrative review illustrates the interdisciplinary aims and challenges of sinonasal reconstructive surgery using flaps or grafts. It is particularly relevant to radiologists and radiation oncologists, at a time when intensity modulated radiotherapy and proton therapy have the potential to further contribute to reduction of morbidity.

## Introduction

Sinonasal cancers represent 3-5% of head and neck cancers with a peak incidence in the 5th to 7th decades and with a male preponderance (1, 2). Histological types are more varied than in the other head and neck cancer locations where squamous cell carcinoma remains the most frequent, followed by adenocarcinoma, melanoma, olfactory neuroblastoma and adenoid cystic carcinoma (1, 3, 4). For a majority of histologic subtypes, surgery, when feasible, is the gold standard of treatment and radiotherapy is usually indicated as an adjuvant modality to optimize local control (5). Surgical techniques have evolved over time, with a clear switch from open to endoscopic approaches in most cases. However, the main surgical challenge remains the same: to preserve critical structures such as the brain, the orbit, the optic nerve and the internal carotid artery. Reconstruction techniques after removal of sinonasal cancers focus on preventing complications associated with the procedure, reducing short- and long-term morbidity (cerebrospinal fluid (CSF) leaks, vascular and functional damages) and preventing aesthetic and functional sequelae (enophthalmos, oronasal fistula). These reconstruction techniques have also significantly changed over the past 2 decades and the reconstructed anatomy is meant to be robust enough to withstand radiotherapy.

Understanding the postoperative imaging changes after head and neck cancer reconstructions is a challenge (6, 7). Sinonasal reconstructions are all the more difficult to analyze on imaging due to their variable anatomy and versatility in the immediate postoperative setting of early complications or routine long-term follow-up. Misinterpretation of post-operative imaging may lead to either a misdiagnosis of tumor recurrence, or on the contrary to delayed diagnosis if the recurrence is mistaken for a reconstruction-related change. Among the daily multidisciplinary challenges of cancer treatment, transfer of surgical advances into radiotherapy planning is little assessed.

Similarly, there are many gaps in knowledge to be filled in the interpretation of postoperative imaging to properly define radiotherapy tumor volumes in the presence of imaging variability imposed by reconstruction. On the other hand, radiotherapy may be responsible for tissue fibrosis or atrophy, the anatomy of the reconstructed region and the functional outcomes may change after radiotherapy compared to surgery alone. Together, these factors impose challenges for all physicians involved in follow-up

This narrative review illustrates the interdisciplinary aims and challenges of sinonasal reconstructive surgery using flaps or grafts. It is particularly relevant to radiologists and radiation oncologists, at a time when intensity modulated radiotherapy (IMRT) and proton therapy have the potential to further contribute to reduction of morbidity following on strategy initiated by surgeons.

## From open to endoscopic endonasal surgery

Open surgery with transfacial and craniofacial resection has long been the only method for the surgical treatment of sinonasal tumors, historically. Since the emergence of endoscopic endonasal surgery, open surgical techniques were progressively being replaced unless involvement of the lateral or anterior wall of the frontal sinus or the anterior wall of the maxillary sinus, for example, are involved (8–10). Open surgery is also used in cases of bone invasion or subcutaneous soft tissue involvement, requiring wide excision.

Initially reserved for treatment of chronic rhinosinusitis and functional surgeries limited to the nasal cavities and paranasal sinuses, endoscopic endonasal surgery has evolved with the development of endoscopic skull base approaches and transnasal craniotomy techniques and has since been used for malignant tumors (11–13). Endoscopic approaches were first described for intestinal-type adenocarcinoma (ITAC), then for adenoid cystic carcinomas (14), melanomas (15) and are now implemented regardless of histology. For malignant tumors, the resection uses a centripetal technique. Tumor debulking is primarily performed in order to identify the tumor attachment base (often called pedicle, although this should not be confused for vascular pedicle). Then, clinically uninvolved sinuses preoperatively are opened to allow visualization of the medial orbits, nasofrontal recesses, and sphenoid sinuses. Bony landmarks (optic canals, carotid canals) are identified. Finally, centripetal tumor resection is performed as well as resection of a safety plane (additional margin) where tumor was present. Iterative frozen section biopsy is performed in macroscopically healthy tissue remote from the tumor throughout the procedure to ensure comprehensive resection (16). The operative report should accurately describe the gross tumor, its base and extensions as well as resection areas depending on whether margins are possibly involved (and to be confirmed with pathology report of similar granularity/accuracy).

Open or endoscopic endonasal techniques may use different reconstruction techniques. Local grafts or flaps are used in endoscopic endonasal reconstruction whereas larger defects may be repaired in open approaches. If reconstruction is performed, it should be described in terms of tissue components, insertions and vascularization. Peroperative and immediate postoperative complications should also be reported.

## Why is reconstruction necessary after sinonasal cancer surgery?

When the tumor involves the skull base, a post-operative CSF leak may occur in 0.5 to 5% of surgical series (8, 17, 18). This risk depends mostly on the location and the size of the defect (19). Several complications can occur as a result of CSF leak including meningitis, empyema, brain abscess, decreased cranial pressure, brain herniation, and death (20). Reconstructive surgery therefore aims at restoring the barrier between the subdural space and sinonasal cavities to avoid postoperative CSF leak. The development of duraplasty techniques (multilayered reconstruction with grafts or vascularized flaps) allowed a large reduction in morbidity and mortality. In recent series, the overall success of CSF leak repair with endonasal flaps is now about 95% (21). A raised intracranial pressure is the main factor associated with failure of duraplasty but other factors depending on the location and size of the defect, or the pathology itself (e.g.: craniopharyngiomas) must be taken into account (22). A special attention to the quality of CSF leak closure is of paramount importance in these situations. A history of radiotherapy (poor tissue healing) or prior surgery with compromise of local vascularized tissue reconstructive options make reconstruction techniques more difficult (23).

In case of intraconal tumor extension and invasion of the infrastructure of the maxilla, the oncologic resection requires open approach with orbital exenteration and open maxillectomy, respectively. In these situations, a reconstruction for functional and aesthetic purposes is mandatory. These reconstructions are mainly performed using free flaps providing bony components (24, 25). Bony reconstructions can further allow the installation of nasal or orbital epithesis, or the implementation of a good dental rehabilitation in maxillary reconstructions. In situations where bone or soft tissue are resected, free flap reconstruction often necessitates a bone flap to reconstruct the maxillary defect. The subcutaneous soft tissues and the cutaneous part are often reconstructed by chimeric flaps (associating independent components and each having their own vascularization) or composite flaps (associating independent components and having the same vascularization).

When critical anatomical structures such as the internal carotid artery, the optic nerve or the clivus are exposed to air or saliva postoperatively, reconstructive surgery aims to protect these structures from damages, ideally with a vascularized flap as it improves the effectiveness of the reconstruction in the long term (26, 27). This is particularly important when postoperative radiotherapy is considered (26–28).

## Type of reconstructions in sinonasal tumors surgery 

Tissues used for reconstruction surgery include grafts and flaps (**Table 1**). Grafts are tissues harvested locally (nasal mucopericondrium/mucoperiostium) or from another site (abdominal fat, fascia lata). Grafts do not bring their own blood supply and may indeed not need vascularization as their main aim is to protect an anatomic area from by use of solid coverage. Graft living tissues may turn into fibrous inert tissues. They are usually harvested from the patient, however occasionally, the reconstruction will not use autologous tissues. Flaps are tissues harvested from a donor site and moved to the recipient site with their blood vessels. Flaps can be harvested locally (nasal fossae), regionally from an adjacent region (temporal fossa, face) or at distant sites (free flaps requiring microvascular anastomosis).

**Table 1:** Types of various surgical flaps used in sinonasal cancer surgery, postoperative & long-term follow-up imaging features.

	Type of reconstruction	Postoperative imaging Features	Long-term evolution
**Dural plasty**	Fascia lata graft	MRI : T1-Hypointense signal Contrast enhancement variable Rarely individualizable	Involution
**Local pedicled flaps**	Nasoseptal flap	CT: Rarely individualizable from adjacent mucosa MRI : isointense T1 and T2, C-shaped	Involution
	Turbinal flap		
**Regional pedicled flaps**	Temporal fascia flap	MRI: Alternation of hypo- hyperintense connective tissues on T2-weighted sequences	Involution
	Pericranial flap		
**Free flaps**	Radial forearm flap	CT: Hyperdensity tissues of skin and subcutaneous tissues	Volume remains stable
	Anterolateral thigh flap	CT: Central fatty component MRI: Fatty component	Fatty transformation Volume remains stable
	Fibula free flap Scapula free flap Iliac crest free flap	CT: Bony part clearly identified Cortical bone (CT), trabecular bone (MRI)	Bone consolidation Risk of osteoradionecrosis, wound breakdown, plate extrusion

### Free grafts

Free grafts are frequently used for duraplasty. Free grafts can be used in combination with another free graft [e.g. fascia lata with a piece of cartilage (29)], or in combination with a local pedicled flap. In post-operative imaging (**Table 1**), the different components of the grafts may no longer be individualized and a hypointense signal without contrast enhancement is observed between the two parts of the bone defect.

#### Autologous fascia lata graft

**Figure 1:**
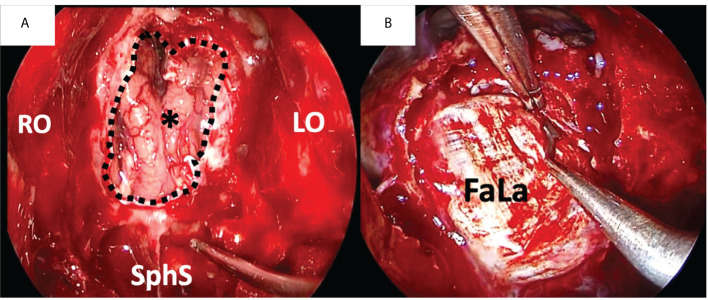
Duraplasty with fascia lata (FaLa) after endoscopic transnasal craniectomy for an intestinal type adenocarcinoma. **(A)** Endoscopic view of the surgical field after tumor removal: the right and left orbital walls (RO and LO, respectively), the sphenoid sinus (SphS) and the frontal lobe (asterisk) are exposed; the dotted line marks the limits of dural resection. **(B)** The first layer of fascia lata is placed intradurally to obtain watertight closure.

The fascia lata graft is a non-vascularized connective tissue, very thin (1-2mm), harvested from the patient’s thigh and positioned to close the defect in a single or multilayer fashion depending on surgeon preferences (**Figure 1**). This technique is effective and safe with 3% donor site morbidity (30, 31). Using facia lata graft protect from the increased risk of CSF leak reconstruction after radiotherapy (31). In the long term, fibrosis of the various components used for reconstruction appears.

#### Fat graft

**Figure 2:**
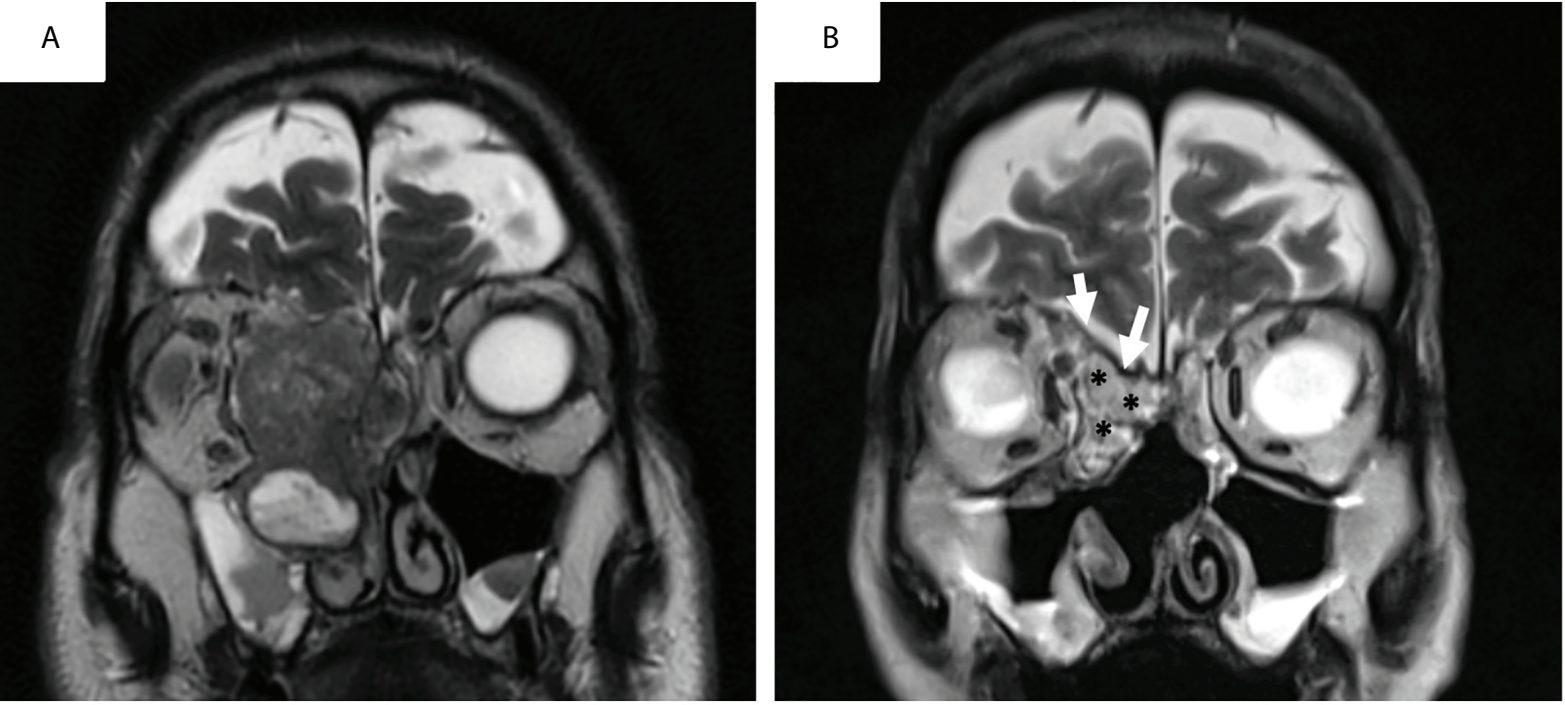
Pre-operative **(A)** and early post-operative aspect (postoperative day5) of a skull base reconstruction with fascia-lata and abdominal fat graft **(B)** for an intestinal type adenocarcinoma on T2-weighted MRI sequences.

Adult abdominal fat, which is known to contain pluripotent stem cells, is frequently used to repair limited anterior skull base defects. Fat lobules are taken from the abdomen by a sub-umbilical incision or from the subcutis of the tight incision in case of simultaneous need of fascia lata. In large skull base defects, fat grafts can’t be left in a place in contact with air flow inside the nose because of the risk of liponecrosis, increased by radiotherapy. It is then recommended to cover fat graft with mucosal graft or a pedicle flap in order to avoid this complication. This technique is safe and has a low rate of complications. In particular, an increase in carcinological risk has never been described following stimulation by stem cells derived from adipocytes (32). In the long term, fat atrophy is observed (32). In early postoperative, fatty components can be visualized in hypersignal in T1-weighted and T2-weighted MRI sequences, however this examination is rarely performed in the very early postoperative period (**Figure 2**).

#### Mucosal graft

Mucosal grafts are pieces of mucosa, harvested from the nasal cavity floor, middle or inferior turbinate or nasal septum in a quick and minimally morbid way (33) that can be used to reconstruct limited skull base defects in combination with other free grafts.

#### Cartilaginous or bone grafts

Cartilaginous grafts, harvested from the septal cartilage can be used as a first layer of closure and fashioned to the size of defect. Bone graft, harvested from the vomer, could also be used in this case. Although less effective than a fascia lata, this type of reconstruction can be useful for limited skull base defects and small orbital defects (34).

### Local pedicled flaps

**Figure 3:**
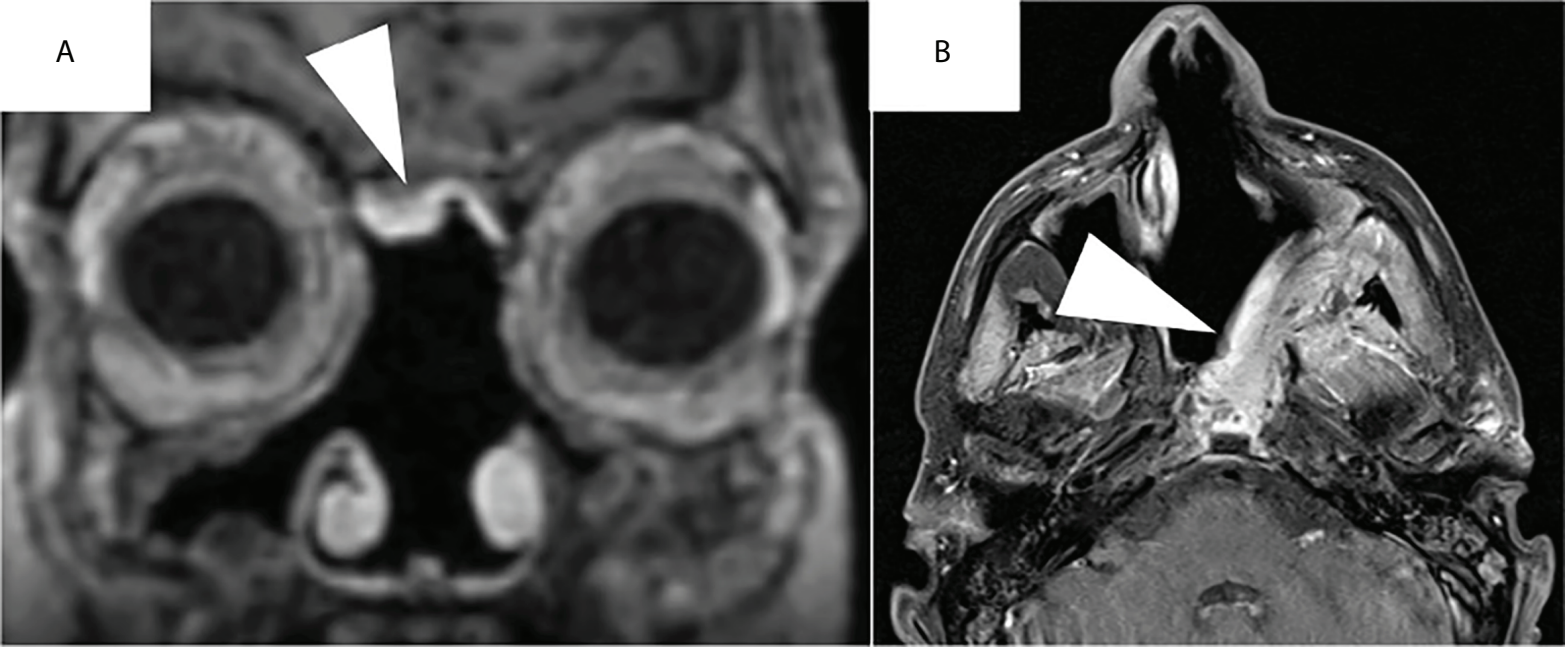
Post-operative aspect of a nasoseptal flap **(A)** and of a left superficial temporoparietal fascia flap **(B)** on contrast-enhanced T1-weighted MRI sequences. Note the hyperintense aspect of the flaps after contrast enhancement (white arrowheads).

The use of free grafts makes it possible to cover a loss of substance. However, its lifespan is limited due to the absence of vascular blood supply. The use of local or regional pedicled flaps ensures vascularization and improves the effectiveness of the flap over the long term. In postoperative in CT-scan, local flaps are rarely individualizable from the adjacent mucosa. In MRI, local flaps have a C-shaped configuration within the operative defect, isointense on T1-weighted and T2-weighted images on both immediate and delayed MRI. After contrast enhancement, local flaps have an hyperintense aspect (**Figure 3**).

#### Nasoseptal flap

The pedicled nasoseptal flap (NSF) is the first choice for most skull base reconstructions thanks to its large surface, ease of harvesting and versatile rotational angle of the pedicle. The use of this flap as described by Hadad et al. (35), has diminished the risk of postoperative CSF leaks and morbidity associated with extended endoscopic skull base approaches (36, 37). NSF is a mucoperiosteal flap pedicled on the posterior nasoseptal artery. It could be elevated to repair the skull base defect or to cover an exposed structure (e.g. carotid artey).

#### Turbinal flap

The turbinal flap is a mucoperiostal pedicled flap based on the middle and superior turbinates and pedicled on the ethmoidal artery system. Compared with other endonasal flaps, the turbinal flap has a double pedicle from the anterior and posterior ethmoidal arteries, which confers abundant blood supply. A vertical incision is performed at the middle turbinate anterior edge and a subperiostal dissection is carried out on the lateral side of the middle turbinate until the mucoperiostal flap is isolated from the bony framework and the flap elevated. This flap is adequate to cover defects along the entire ethmoid roof up to a size of 8cm^2^. It can also cover the medial orbital wall and medial portion of the roof of maxillary sinus (38).

#### Septal flip flap

The septal flip flap is a mucoperiostal pedicled flap harvested from the contralateral nasal septum based on the septal branches of the anterior and posterior ethmoidal arteries and can be rotated to resurface an anterior skull base defect (39). This flap provides vascularized mucosal coverage extending from the frontal recess back to the planum sphenoidalis (40).

#### Lateral nasal wall flap

The lateral nasal wall flap is a mucoperiostal pedicled flap harvested from the lateral nasal wall. It is based on a rich vascular supply based on an anterior pedicle comprising branches of the facial (angular and lateral nasal) and ethmoidal arteries. This flap is useful when an anterior pedicle is needed and when the septum is absent (41).

### Regional pedicled flaps

When a local pedicled flap cannot be used due to a too large defect, an unavailability of nasal mucosa in cases of salvage reconstructions or a prior irradiation which causes an unfavorable condition for local flap viability, a regional pedicled flap can be used. These flaps allow to cover large skull base defects and vascular exposures in the nasal cavities. In postoperative imaging, local flaps are rarely individualizable in CT-scan. Frequently composed of connective tissue, they can be distinguished in T2-weighted MRI sequences. Regional flaps have also a C-shaped configuration within the operative defect, isointense on T1-weighted and T2-weighted images and an hyperintense aspect after contrast enhancement.

#### Superficial temporoparietal fascia flap

**Figure 4:**
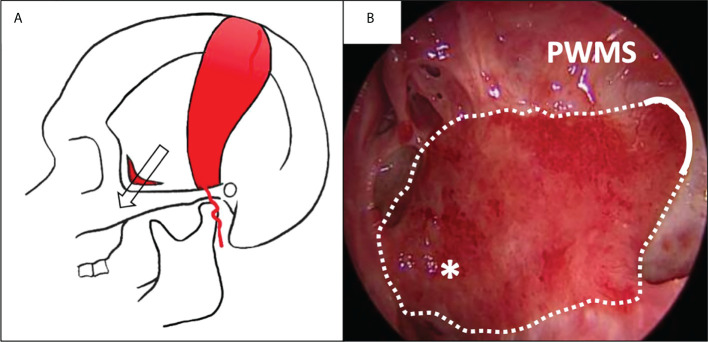
Covering of the lateral nasopharyngeal wall with a superficial temporoparietal fascia flap (sTPFF) after left endoscopic rhinopharyngectomy for recurrent nasopharyngeal carcinoma. **(A)** the sTPFF is pedicled on the superficial temporal artery; it is transposed in the nasal cavity through a temporal/infratemporal fossa tunnel (arrow). **(B)** Post-operative endoscopic view after 2 months: the flap (dotted line) has been introduced through an opening in the posterior wall of the left maxillary sinus (PWMS) and covers the lateral wall of the nasopharynx (asterisk), thus protecting the parapharyngeal internal carotid artery.

The superficial temporoparietal fascia flap (TPFF) is a connective tissue flap vascularized by the superficial temporal artery, which has a larger diameter than the ethmoidal or sphenopalatine arteries and is usually spared by external radiations in case of prior radiotherapy (42, 43). Its properties, such as thinness, foldability and its long pedicle make it a versatile flap for reconstruction of various defects of the skull base, both in adult and children. It is a good reconstructive option for defects of the middle and posterior fossa (44). It is less used for anterior skull base repairs because of the orientation of its pedicle (45). Its transposition into the nasal cavity through a hemi-coronal approach is frequently performed through a temporal-infratemporal tunnel (42). Other way for TPFF transposition were more recently described like the supraorbital epidural corridor (46). It represents a safe and effective technique with morbidity limited to potential alopecia, facial nerve damage and scalp necrosis (**Figure 4**).

#### Pericranial flap

**Figure 5:**
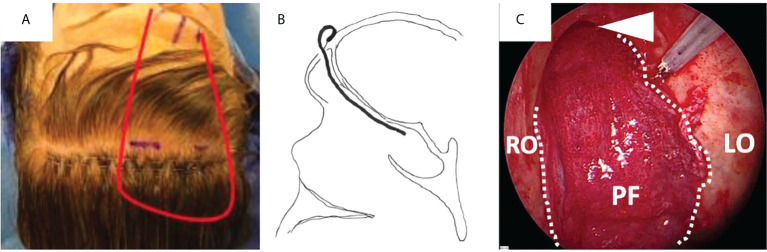
Covering of the anterior skull base with a pericranial flap (PF) after endoscopic transnasal craniectomy for an olfactory neuroblastoma in a previously irradiated patient. **(A)** Lining of the pericranial flap (red line) pedicled on the left supraorbital and supratrochlear pedicles. **(B)** The flap is placed against the whole anterior skull base through an opening in the superior aspect of the anterior wall of the frontal sinus. **(C)** Final endoscopic view, with the flap covering the whole anterior skull base, from the right to the left orbit (RO and LO, respectively) laterally and from the frontal sinus (white arrow head) to the sellar region posteriorly.

The pericranial flap is a versatile and robust pedicled flap used for skull base reconstruction and has been the workhorse of anterior skull base reconstruction for several decades. It is taken from the aponeurotic system between the frontalis and occipitalis muscles. It receives its blood supply from the supraorbital and supratrochlear vessels, terminal branches of the ophthalmic artery (47). Initially requiring a coronal incision, minimal invasive techniques for harvesting the pericranial flap were gradually developed with small scalp incision, endoscopic dissection and minimal cosmetic bone defect to gain access to the nasal aspect of the skull base (48, 49). It can cover from the anterior cranial fossa as far as the sella, without reaching posterior cranial base defects (44). The thickness of the flap remains relatively stable over time for most patients even following radiotherapy (50, 51). Although there is a risk of obstruction of the frontal sinuses, passage of the flap in the midline in conjunction with complete removal of the floor of the frontal sinuses maintains a lateral drainage pathway (52) (**Figure 5**).

### Free flaps

When the size and location of the skull base defect to be repaired exceeds the excursion limits of pedicled flaps, free flaps can be considered. They are mainly used during open sinus surgeries, leading to an important facial defect. Endoscopic assisted inset of the free flap in the anterior skull base is also feasible in selected cases (53).

#### Radial forearm flap

The radial forearm flap has been shown to be a safe free flap when used for both head and neck as well as skull base reconstruction (54). This flap is composed with skin, fascia, fat (2-10mm layer) and muscle. Its skin paddle is taken from the distal part of the forearm, supplied by the radial artery and vein. This flap has a long pedicle which allows for usage of neck vessels as recipient vessels. It is exclusively composed of skin and fascia and is relatively thin. Due to its pliability, it is versatile for skull base defect reconstructions. It can also be folded back on itself to create more volume where needed (55). This makes it an ideal choice of flap when the defect is small, but reliable skull base or dural closure is required (56). However, reconstruction of the donor site requires a thin skin graft, often taken from the thigh, which can be a secondary source of pain and skin discoloration on healing (57). In postoperative imaging, the hyperdense tissues of the skin and subcutaneous tissues are thin and located on the mucosal side of the flap. Postoperative radiotherapy might reduce soft-tissue flap versatility due to fibrosis but in the lack of correlative studies, it remains controversial.

#### Anterolateral thigh flap

The anterolateral thigh flap is frequently used when a free flap with both muscle and adipose bulk is desired (58). This flap offers versatility in the components taken when harvesting the flap as well as creative inset options. It is mostly composed of fat and skin and can be harvested with or without muscle depending on the volume needed (59). The volume of transplanted fat vary according to the Body Mass Index of the patient (60). Its vascularization is supplied by a descending branch of lateral circumflex artery. Two veins accompany the artery and then merge into one at profundal femoral vein junction. It could be used for full-thickness nasal defect reconstructions, mostly orbital walls or total maxillectomy defects, with good functional results (61, 62). In postoperative imaging, it is easily differentiated from adjacent tissues, thanks to the hypodense central fatty component, which can reach 1 to 3cm. Although it is frequently observed a fatty atrophy, this flap provides relatively stable volume maintenance over time even after postoperative radiation and the impact of flap atrophy on functional deterioration or the need for surgical overcompensation are both controversial from surgical and radiotherapy standpoints (60).

#### Rectus abdominis free flap

Rectus abdominis free flap is a versatile flap that is well suited to a variety of reconstructive defects in the head and neck including maxillary or orbital defects. Vascularized by the deep inferior epigastric artery, this flap can be harvested with or without a skin paddle with a long pedicle. The versatility of this donor site is due to the ability to transfer large areas of skin with varying thicknesses and varying amounts of underlying muscle (63).

#### Latissimus dorsi free flap

The latissimus dorsi free flap can be used for maxillary or orbital reconstructions. It is harvested from the thoracodorsal artery (terminal branch of the subscapular artery) as a simple muscle flap or a musculocutaneous flap. Due to the size of the muscle it can be used to cover large maxillary or orbital defects (64).

#### Osteocutaneous free flaps

Restoring the facial contour based on the concept of facial buttress reconstruction improves aesthetic outcomes (65). Osteocutaneous free flaps should be used in case of significant orbital or maxillary defects (66).

The fibula free flap is the main composite bone flap used in head and neck cancer bone reconstruction. It can be used as an osteo-cutaneous flap or a bone flap without skin, and its fatty part is very thin. It provides a large amount of bone and a long pedicle. It is vascularized by interosseous and segmental perforators.

Scapula free flap reconstruction is also versatile in orbitomaxillary reconstruction (67). Scapula free flap can be harvested as a chimeric flap with bone, muscle and skin all harvested on separate branches from the subscapular system (circumflex scapular artery). The scapula free flap provides large flat bone and is surrounded by fat (hypodense) and muscle (homogenous moderate density compared to mucosa). One of the advantages of the scapula free flap over the fibula free flap is that the subscapular arterial system is usually protected from atherosclerotic disease as compared to the fibular vascular system (68).

Iliac crest free flap is useful for composite bone and soft tissue reconstructions. It is harvested on the deep circumflex iliac artery and offers a large quantity of high-quality bone, necessary for dental rehabilitation with osseointegrated implants. It has however a relatively short pedicle length (8-10 cm) and induces an important donor site morbidity (69).

Bony reconstructions require fixation with plates and screws and utilization of mirror image based virtual surgical planning and a 3D-printing guides improves aesthetics and cosmetics results (70, 71). Long-term complications occurred in about 15% of cases and concern principally wound breakdown, plate extrusion or osteoradionecrosis. Bone flaps are known to be at higher risk for osteoradionecrosis than the native bone (7). Dose backscatter from plates and screws might result in increased dose to the native and flap segments at their junction and increased risk of osteonecrosis, but lack of data prevents any estimate of the risk. There also might be an increased risk of osteoradionecrosis with 3D-printed piecemeal osteotomies. These bone fragments are deperiosted and the frail vascular suppliance might further contribute to the vascular-related risk factor for osteoradionecrosis. Prior radiotherapy predisposes patients to long-term complications (72).

Success rates of microvascular free flap reconstruction approach 95% and the main complication of these reconstruction is flap failure. It must be detected early and managed efficiently because of the short window of opportunity for flap salvage. Free flap failure may primarily be due to thrombosis. Arterial thrombosis occurs early in the immediate postoperative setting whereas venous thrombosis occurs later, frequently after 72h. Necrosis of free flap is rarely due to radiation-induced damage of vascular anastomosis or thrombosis but often occurs in the early postoperative period and could be caused by the vessel quality, comorbidities or technical procedures (7). Perforator flaps would seem to be more robust to radiotherapy. However, experience is currently mostly limited to skin cancer and data on perforator flaps in mucosal head and neck cancers are needed.

## Which reconstruction for which situation? 

**Figure 6:**
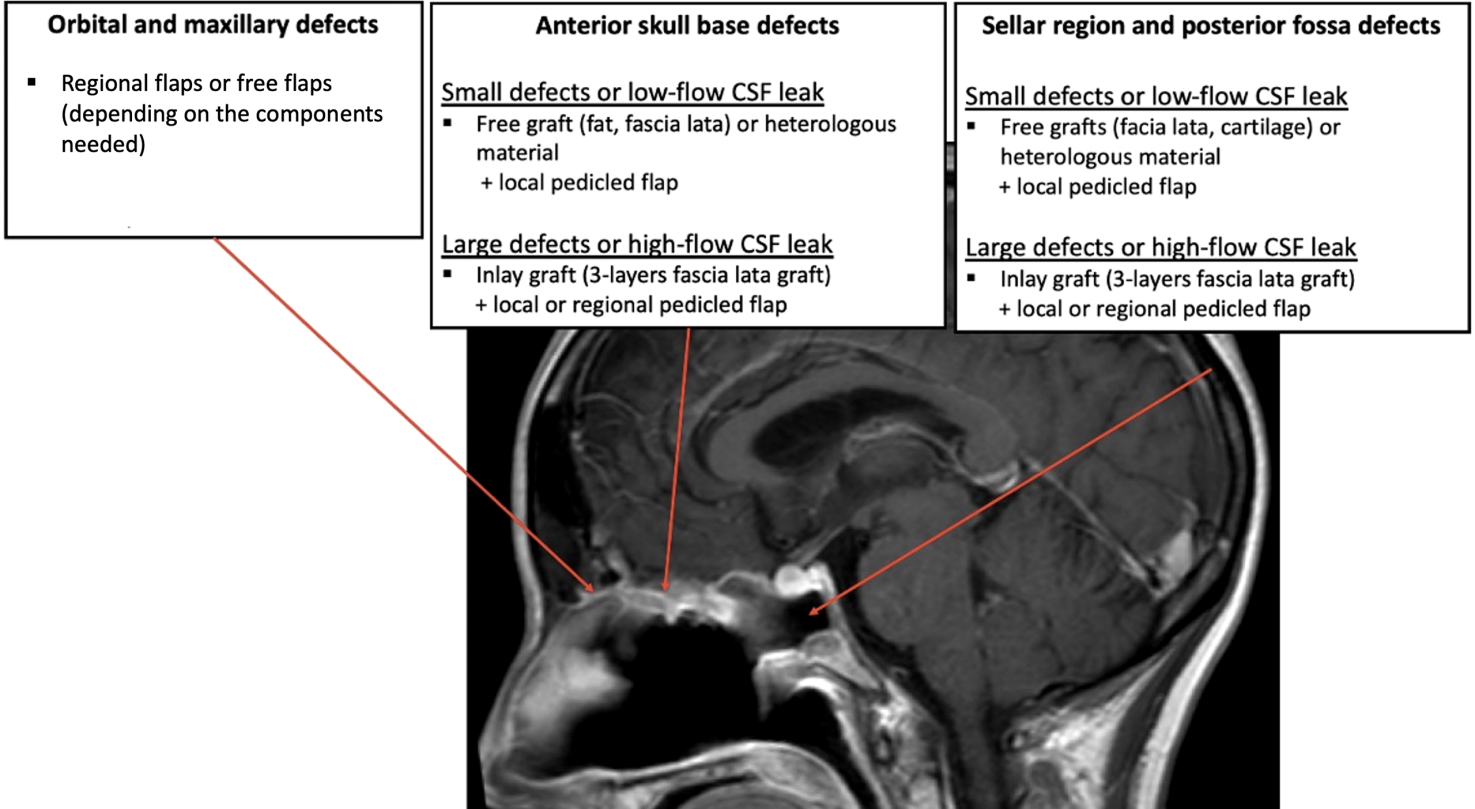
A proposition for reconstruction strategies according to the location and size of the defect after surgical removal of the tumor.

In the literature, there is no consensus concerning a reconstruction choice algorithm. Some parameters must be taken into account when making the decision: the size and the location of the defect, the materials and flaps available locally, the need for postoperative radiotherapy (or past history of radiotherapy) and the surgeon’s expertise and choices. The most widespread indications described in the literature are reported below (**Figure 6**).

### Skull base defects requiring duraplasty

Anterior fossa defects, excepted limited defects (<1 cm), are best repaired with inlay grafts, for example fat or fascia lata, because the pressure from the brain helps maintaining the material in position and avoid its endonasal migration. A 3-layer reconstruction with the iliotibial tract of the fascia lata is usually necessary in case of large defect (>3 cm) after transnasal craniectomy (30). The skull base primary reconstruction can then be covered with a vascularized mucosal local flap to protect the graft and facilitate healing (30). Indeed, a flap does not replace the primary dural plasty and watertight closure must be obtained before placing the flap on the reconstruction. For very small defects, for example in the area of the cribriform plate, it is possible to use a free graft (or even heterologous material such as the Tachosil^®^ sealant) in an onlay position, ideally covered by a local pedicled flap.

Defects of the sellar region or of the clivus are more difficult to close because of the high intracranial pressure associated with the proximity to the cisterns and ventricles. Small defects (<1 cm) may be repaired using multilayered free grafts, for example fascia lata and cartilage in the “gasket seal” technique with high success rates (29). Large dural defects (>3 cm) involving wide dural and arachnoid dissection and high-flow CSF leaks should preferably reconstructed with multilayer duraplasty covered by a vascularized flap (73, 74). To better guide the choice of the surgeon on the type of reconstruction and the type of flap to be used, surgical base defects could be predicted, with the help of computer tools, on preoperative CT-scans (75).

When a multilayer reconstruction is performed, the different components of the reconstruction are maintained in their final position by biological glue and/or packing. The flaps are very rarely maintained by sutures or staples.

### Covering of critical structures

In case of exposure or critical structures (internal carotid artery, optic nerve, clivus bone, or even intact dura), the best way to prevent post-operative complications is to cover the exposed structure with a vascularized flap. Depending on the location of the defect and on the available flaps, local pedicled flaps or regional pedicled flaps can be used in these situations.

### Bony reconstruction

When an open technique is performed with bony defects (infrastructure of the maxilla, orbit), the extent of injury needs to be assessed and reconstructed accordingly. Free flaps providing bone component should be considered and utilized whenever possible as they provide excellent functional and aesthetics results (76). For these reconstructions, flaps used are increasingly versatile by providing muscle, fat and bone components depending on the subunits to be reconstructed.

### Past history of radiation therapy

Skull base tumor recurrence after radiation therapy is difficult to resect and reconstruct. In this case, multilayer reconstruction with vascularized flap helps to optimize healing of the operative field.

## Post-operative imaging after skull base reconstruction

Because of a frequency of 30 to 50% of local recurrence after surgery for malignant sinonasal tumors (3), it is necessary to detect early a possible recurrence. This detection is both clinical and paraclinical using imaging assessments. Postoperative imaging evaluation is therefore one of the cornerstones of monitoring patients undergoing endoscopic sinonasal surgery for malignant tumors. The detection of local recurrences is difficult in this context since it is necessary to integrate the post-operative changes related to the removal of the tumor but also the elements of reconstruction (grafts and flaps). Performing post-operative imaging is essential since it serves as a baseline and integrates post-operative changes that will be key to the interpretation of further local changes and to better detect subsequent recurrences. In this sense, MRI is the most appropriate imaging examination since it makes it possible to detect the various elements of reconstruction and allows most of the time to distinguish between a recurrence and post-therapeutic changes. PET/CT is often useful to visualize a possible recurrence after oncological surgery. However, the inflammatory environment of the posttreatment in sinonasal cavity leads to a high number of false positives. PET/CT has a worse specificity and positive predictive value in sinonasal malignancy than in head and neck malignancy overall, especially in the early posttreatment period (77). There is no optimal timeframe for performing the first postoperative imaging. We can however think that an MRI performed between 3 and 6 months post-operative is mandatory. In order to perform a proper assessment of the radiological findings, it is essential to know the radiological variations of sinonasal reconstructions during the healing process.

In free grafts and local or regional flaps reconstructions, the different components of the reconstruction (grafts and flaps) may no longer be individualized in CT-scan and a hypodense signal without contrast enhancement is observed between the two parts of the bone defect. In MRI, local and regional flaps have a C-shaped configuration within the operative defect, isointense on T1-weighted and T2-weighted images on both immediate and delayed MRI. After contrast enhancement, it is observed an hyperintense aspect (78). The presence of fatty components in the reconstruction technique can be confirmed in MRI with the fat suppression technique. Flaps composed of connective tissues can be distinguished in T2-weighted sequences, alternating hypointense-hyperintense appearance representing the layers (49). Thickening of the sinonasal mucosa is typical after surgical and radiation treatment. Inflammation manifests as ballooning of the mucosa with hyperintense T2 signal. After contrast enhancement, the epithelial lining enhances whereas the underlying edematous submucosa does not. Graft and flap reconstructive surgery and associated synthetic/metallic materials substantially impact surveillance. Better awareness of radiologists and detailed description of their procedure by surgeons as well as interdisciplinary discussions are critical to the detection of complications and recurrences during follow up.

After radiotherapy, such changes are seen immediately in early follow-up studies and may persist as long as 30 months. Chronic inflammation of the mucosa may also favor the formation of synechiae between bone or mucosal structures and the flap. In free flaps reconstructions, bone flaps can be easily distinguished from the native bone by the rupture of continuity of the cortical. Spontaneous bone resorption occurs over time by about 0.2 mm/year in the native mandible and can be applied by analogy to the maxillary. This resorption occurs in transplanted bone to a lesser degree (79). Fixation screws and plates at the flap-native bone interface can be responsible for metallic artifacts and backscatter radiation, which might increase their susceptibility to osteoradionecrosis (80). In these reconstructions, numerous clips are usually present at the vascular pedicle or at the anastomosis between the flap and the tumor bed. They are rarely used to indicate areas of dubious margins and thanks to their small size, they little degrade image quality (7).

Although success rates of microvascular free flap reconstruction approach 95% (81), the vascular supply may be compromised in the early postoperative period because of injury or compression. Free flaps for extended maxillofacial reconstruction are at risk of necrosis in connection with vascular phenomena (82). To evaluate the vitality of these flaps, dynamic contrast-enhanced CT-scan and MRI can be performed. The heterogenous contrast enhancement is difficult to correlate with flap failure because there is overlap in the imaging appearance of an enhancing flap and granulation tissue, particularly of the delayer scans. Immediate postoperative imaging are more helpful to evaluate the enhancement of the flap because there would be less time for granulation tissue to form (76).

As with other flaps of the head and neck, the fatty part within the reconstructions is likely to atrophy. The fatty part is visualized hypointense in postoperative CT scan and with the fat suppression technique in postoperative MRI. This atrophy is all the more important as postoperative radiotherapy is routinely performed for sinonasal carcinomas. Although flap fibrosis is a functional disadvantage in the context of extensive reconstruction, it is also the desired objective in the case of CSF leak repair.

Recent imaging techniques, as Dual-Energy Computed Tomography (DECT) provides high overall image quality for tumor delineation in head and neck imaging and reduces beam hardening artifacts. After sinonasal reconstruction, 3D volume rendering reconstruction allows virtual visualization of the flap and the feeding vessels to be spared (83).

## Impact of sinonasal reconstruction on radiotherapy planning

Sinonasal flap reconstructive surgery does substantially modify the anatomy. During radiotherapy planning, ectopic fat or fascia/skin of graft can be seen as thin hypodense or spontaneously hyperdense layers at the surgical borders of the operative bed. Ectopic flap tissues can be identified by their components: ectopic fat well seen as hypodense central areas, “geometric” bone with metallic fixation devices (and often surrounding artefacts that render delineation even more complex), iso/slightly hyperdense muscle/skin/fascia components.

**Figure 7:**
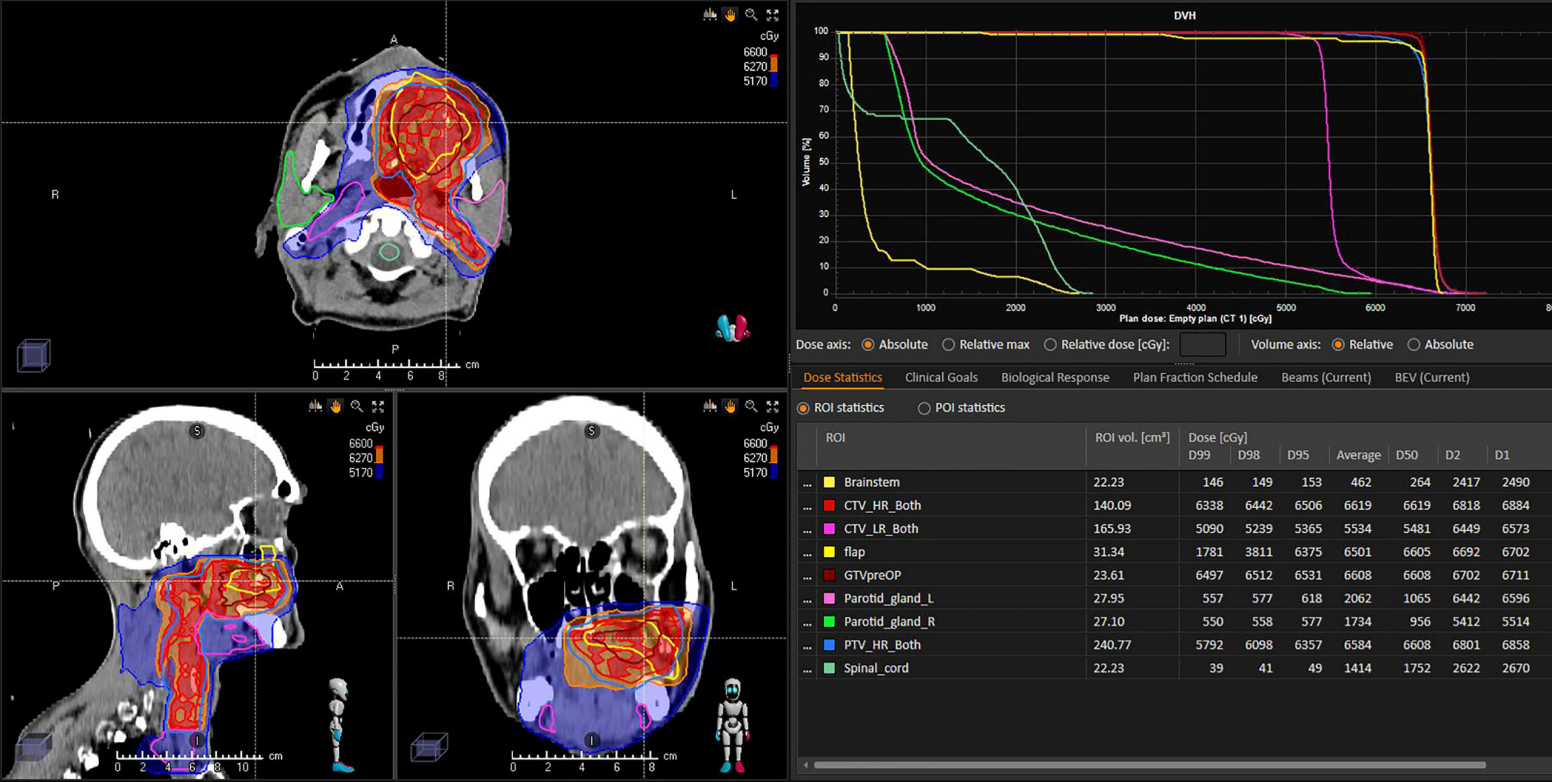
Postoperative radiotherapy planning of a left palatine squamous cell carcinoma following reconstructive surgery with a soft-tissue forearm flap. The flap (yellow line) was not delineated for RT planning and has been delineated a posteriori independently of referring radiation oncologist and blind to CTV delineation. Analysis of radiation dose distribution suggests that the flap was considered as a target volume and received the highest dose prescription level (66 Gy, within red 62.7Gy isodose).

As reported in conventional head and neck carcinomas, radiotherapy planning is currently blind to surgical changes and flaps are not delineated per lack of knowledge on their aspect on imaging (7). In head and neck free flaps, the concept of flap sparing was however published by Bittermann yet neither implemented nor challenged in practice since (84). Flap changes may occur spontaneously or due to radiotherapy. However, several surgical series have reported that radiotherapy may deteriorate the results of reconstructive surgery in terms of functional outcomes. To assess dose-effect relationships of flaps and functional outcomes in comparison with surgery only, radiation oncologists will have to be able to delineate flaps on their planning CT or MRI as was done in conventional head and neck sites (85). Current practice for free flaps seems to include flaps in the target volumes with clinical target volumes centered on flap hypodense fatty portion (86). For that, a perfect knowledge of the tumor location (tumor implantation, and sites of involved margins), the type of surgery and reconstruction performed with comprehensive operatory reports are critical for the radiation oncologists (**Figure 7**).

Delineation of flaps is critical to better understanding of radiation effects, versus surgery alone. There are hardly any data on flap management during radiotherapy planning. Based on unpublished data, it seems that current practice trends towards including most of the flap volume into the high-risk high-dose radiotherapy volumes. Although one might consider avoiding irradiation of an ectopic, flap, tissue to limit the risk of toxicity and functional deterioration, data are critically lacking. Ambispective studies including flap reconstructive surgery of sinonasal tumors are ongoing.

## Conclusion

The management of defects after sinonasal cancer surgery is a major challenge for patients. Many technical advances in techniques for reconstructing sinonasal defects after sinonasal cancer surgery have been described in recent years. The increasingly common use of vascular pedicled flaps and free flaps, both endonasal and extranasal, is contributing to the meaningful reduction in surgical complications. The impact of reconstruction techniques on postoperative imaging and radiotherapy planning should be taken into account more systematically to assess post-operative radiotherapy effects on reconstructed anatomy, flaps and grafts.

## Author contributions

Manuscript conceptualization: FC and JT. Manuscript writing: all authors. Manuscript correction: all authors. All authors contributed to the article and approved the submitted version.

## Conflict of interest

The authors declare that the research was conducted in the absence of any commercial or financial relationships that could be construed as a potential conflict of interest.

## Publisher’s note

All claims expressed in this article are solely those of the authors and do not necessarily represent those of their affiliated organizations, or those of the publisher, the editors and the reviewers. Any product that may be evaluated in this article, or claim that may be made by its manufacturer, is not guaranteed or endorsed by the publisher.
